# Complex Pulmonary Aspergilloma: Surgical Challenges in a Third World Setting

**DOI:** 10.1155/2018/6570741

**Published:** 2018-01-14

**Authors:** Bernadette Ngo Nonga, Guy Aristide Bang, Bonaventure Jemea, Eric Savom, Perfura Yone, Ngahane Mbatchou, Jean Jacques Ze

**Affiliations:** ^1^Department of Surgery, Faculty of Medicine and Biomedical Sciences, University of Yaoundé I, Yaoundé, Cameroon; ^2^Department of Surgery and Specialties, Service of Anesthesia, Faculty of Medicine and Biomedical Sciences, University of Yaoundé I, Yaoundé, Cameroon; ^3^Department of Internal Medicine, Faculty of Medicine and Biomedical Sciences, University of Yaoundé I, Yaoundé, Cameroon; ^4^Department of Internal Medicine, Faculty of Medicine and Pharmacy, University of Douala, Douala, Cameroon

## Abstract

**Background:**

Surgery for pulmonary aspergilloma (PA), especially complex forms, is greatly challenging in a resource-poor setting such as Cameroon. We report our experience of surgical management of PA in this environment.

**Method:**

We prospectively assessed patients who underwent surgery for PA from January 2012 to May 2015, at the University Hospital Center of Yaoundé. Records were reviewed for demographics, history and physical examinations, radiological findings, surgical procedures, and outcomes. The study has received approval from the institutional ethics committees.

**Results:**

In total, 20 patients (17 males and 3 females (sex ratio, 5.66); mean age, 30 years; range, 23–65 years) with a past history of tuberculosis were assessed. The median follow-up was 21.5 months. The primary symptom was hemoptysis, followed by cough and chest pain. All patients underwent surgical treatment and lung resection. Postoperative complications (bleeding, air leak, empyema, and severe anemia) occurred in 4 patients and 1 patient died. Although 3 patients were lost to follow-up, the survival rate was 80% with improvement of the preoperative symptoms.

**Conclusion:**

Although surgery for complex aspergilloma is very challenging in environments such as ours, we believe that it is the best treatment modality for symptomatic diseases in our setting.

## 1. Introduction

Pulmonary aspergilloma (PA) is a saprophyte that infects preexisting pulmonary cavities producing a fungus ball or mycetoma [1]. The most common preexisting cavity lesion is pulmonary tuberculosis (TB), but it can also be found in healed abscess cavities or in bronchiectasis [2–4]. PA is classified into simple and complex forms; simple forms present as an isolated cavity with thin walls surrounded by normal lung parenchyma, while complex forms are thick-walled cavities surrounded by fibrotic lung tissue with stiff hilar structures, vascular adhesions, and obliteration of the pleural cavity [3].

In Western countries, there has been a recent increase in the prevalence of invasive aspergillosis related to immunosuppression after chemotherapy for cancer or human immunodeficiency virus (HIV) infection [5]. Owing to a high prevalence of TB, related and nonrelated to HIV infection, PA may be more prevalent in developing countries [6]. Studies on PA in sub-Saharan Africa and Cameroon have been rarely reported. Thus, the incidence and prevalence of PA have not been studied and the natural history is poorly understood. It has been reported that PA can be variable in its course, ranging from undergoing spontaneous lysis of the aspergilloma (7–10%) to causing severe life-threatening hemoptysis [7]. Radiologically, it presents as single or multiple ball-like lesions inside a cavity, partially surrounded by a radiolucent air crescent [7].

Whether the optimal treatment of PA is conservative management or radical surgery remains controversial. In many instances, surgery is considered first-line treatment [3, 7–9]. In Cameroon, PA represents a great challenge for the surgeon because of the lack of appropriate infrastructure and surgical materials and supplies and the absence of health insurance for most of the population. No studies have reported cases of PA in Cameroon. Hence, here we report our experience with the surgical treatment of symptomatic and complex aspergilloma in a very resource-poor setting.

## 2. Materials and Methods

### 2.1. Patients

We carried out a prospective study from January 2012 to May 2015 at the University Hospital Center of Yaoundé, Cameroon. All patients with aspergilloma operated on in our center during that period were prospectively registered in a predefined computerized database containing data on demographics, presenting symptoms, tobacco and alcohol abuse, past medical history (TB, HIV infection), radiological findings, preoperative evaluation, operative procedure, postoperative morbidity and mortality, and long-term follow-up.

Hemoptysis was considered moderate if the patient was coughing blood regularly but no more than 300 ml per day. Massive hemoptysis was defined as more than 300 ml of blood coughed per day.

**Figure 1:**
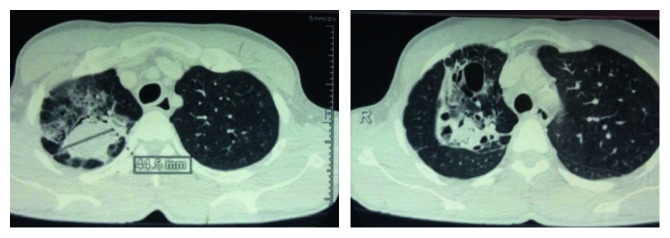
Computed tomography of the chest. A transverse section showing a right-sided complex lesion of the upper lobe adherent to the chest wall.

**Figure 2:**
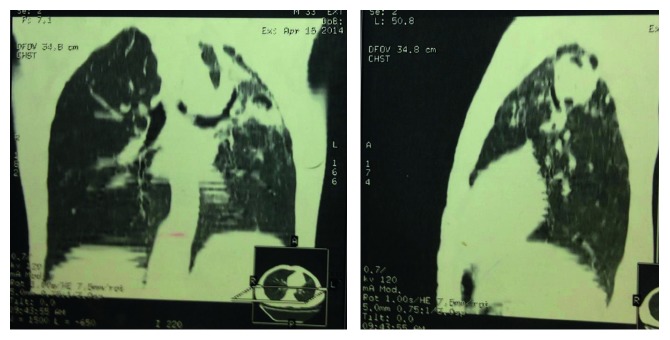
Computed tomography of the chest. A sagittal section showing a left-sided complex lesion of the left upper lobe very close to the aorta.

Patients were referred to us by chest physicians from two cities in Cameroon (Douala and Yaounde). Computed tomography (CT) revealed the presence of a complex aspergilloma (Figures 1 and 2) in all patients. All patients were operated on by the same team.

### 2.2. Preoperative Workup

Complete blood counts, renal and liver function tests, HIV testing, coagulation profile, blood type and crossmatching, electrocardiography, and echocardiography to assess the left and right ventricular function and possible right pulmonary hypertension (which would be a contraindication for surgery, if present) were performed preoperatively. Equipment for pulmonary function tests were not available; hence, we did not perform these tests. Serology tests for *Aspergillus fumigatus* were also not performed owing to the unavailability of testing equipment.

**Figure 3:**
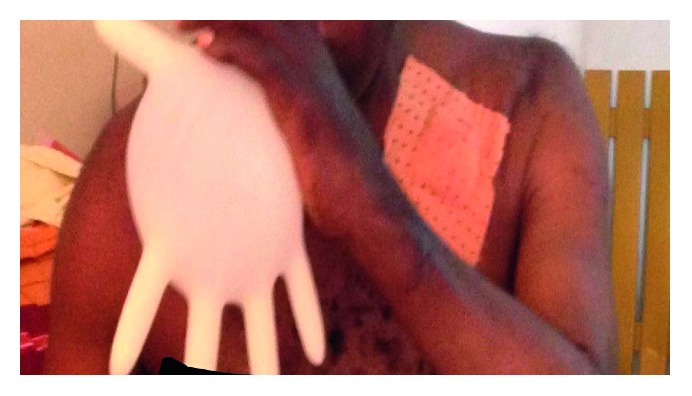
A patient blowing a nonsterile rubber glove used as an incentive spirometer in the postoperative period.

Presurgical preparation included, when possible, respiratory physiotherapy using a glove as an incentive spirometer for patients operated on electively. The same system was used postoperatively (Figure 3).

### 2.3. Procedure

Surgery was performed under general anesthesia. A regular single lumen endotracheal tube was used to achieve selective lung intubation. The tube was pushed into the right main bronchus for surgery involving the left lung and into the left main bronchus for surgery involving the right lung. After induction of anesthesia and satisfactory muscle relaxation, laryngoscopy was performed. To obtain selective right lung intubation, the endotracheal tube was first introduced to the glottis and then further until the balloon reached beyond the vocal cords. The tube was then advanced for 25 cm without rotation before the balloon was inflated. For selective left lung intubation, after the balloon reached beyond the vocal cords, the tube was advanced for 27 cm while rotating it approximately 180° counterclockwise before inflating the balloon. Confirmation was achieved by listening to both lungs using a stethoscope during ventilation.

**Figure 4:**
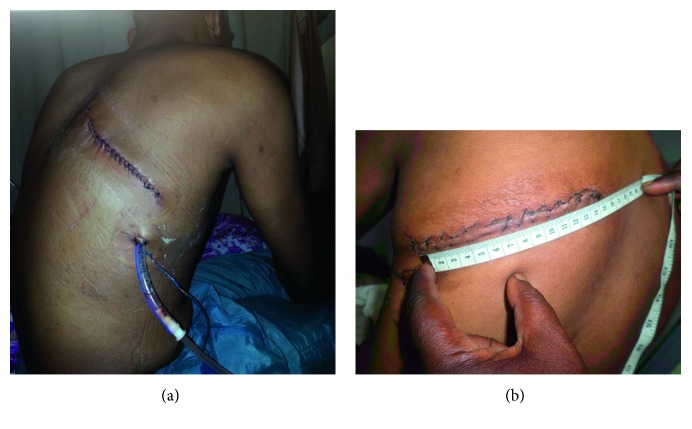
(a) Right muscle sparing posterior thoracotomy for upper aspergilloma on the 5th postoperative day before the drain was removed. The incision is fully posterior. (b) Muscle sparing posterior thoracotomy. A left upper lobectomy for aspergilloma in a 28-year-old man.

Patients were then placed in the lateral decubitus position. Ceftriaxone and metronidazole were used for antibiotic prophylaxis during surgery. We made a small skin incision (14–17 cm; Figures 4 and 4) for posterior muscle sparing thoracotomy to enter the chest cavity through the 5th or 6th intercostal space depending on the type of resection anticipated. We then proceeded to lysis of adhesions and decortication. All the patients had been infected by chronic and destructive TB with subsequent pleural reaction and pachypleuritis. Decortication is a procedure by which fibrotic tissue between the visceral and parietal pleura is removed to free the lung and enable it to fully expand. Chronic pachypleuritis in the affected side was observed in the CT scans (Figures 1 and 2). Complex aspergilloma is characterized by dense and tight adhesions, with lung destruction around the vessels and in the hilum.

Two complications were anticipated when releasing an aspergilloma cavity strongly adherent to the chest wall with no interposition of lung tissue: bleeding due to local rupture of the main cavity vessel and air leakage in situations where the aspergilloma was attached to a major bronchus before this attachment was disrupted. Complex aspergilloma usually destroys the normal lung parenchyma leaving fibrotic and scar tissue centered on a cavity (Figures 1 and 2). It was almost impossible to separate the aspergilloma from the apex of the lung without interfering with its cavity. At times, the aspergilloma was adherent to the apex and medial part of the chest wall, frequently encompassing the phrenic nerve with dense adhesions separating it from the aorta on the left side and superior vena cava medially on the right side. We used low volume ventilation to avoid severe hypoxia after clamping the bronchus to minimize air loss in these complicated cases.

**Figure 5:**
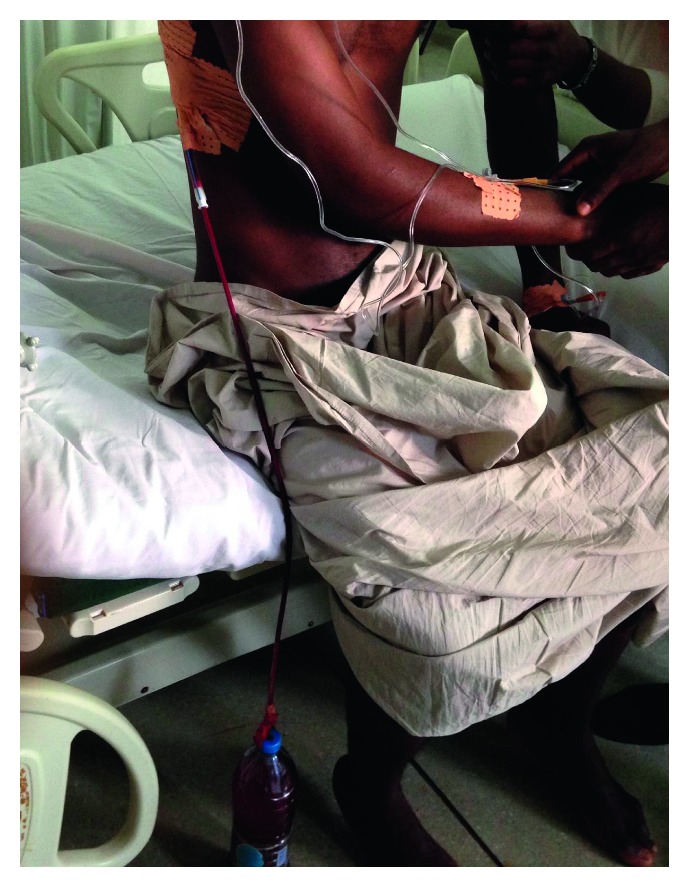
Simple drainage open system used in the immediate postoperative period. A plastic water bottle is connected to the chest drain. Drainage is done by gravity and respiratory movements.

**Figure 6:**
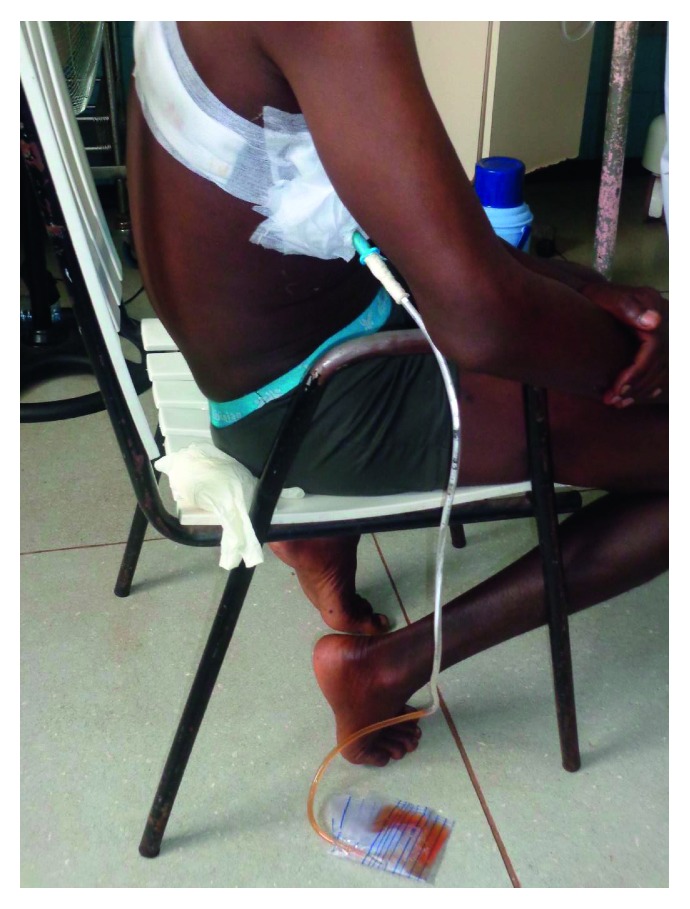
Simple drainage closed system. A urinary collector bag equipped with an antivalve reflux is connected to the drain.

**Figure 7:**
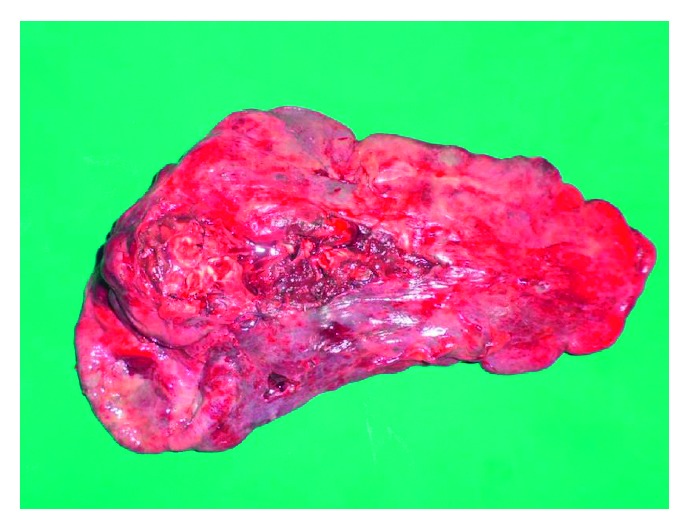
A resected lung's specimen.

**Figure 8:**
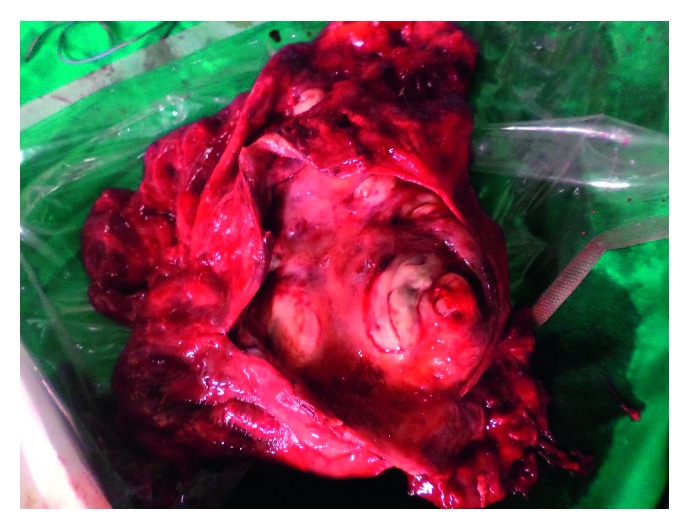
Intraoperative specimen which has been opened. The aspergilloma which was occupying less than 1/3 of the cavity has been removed.

After the lung had been freed, a classical lobectomy or pneumonectomy was then performed. The pulmonary vessels were doubly ligated and the corresponding lobar bronchi were ligated and oversewn. Pneumonectomy was performed if the affected lung was massively destroyed. We did not perform a cavernostomy. After bleeding and air leakage were controlled, we performed a thorough pleural toilet with saline. A simple surgical drainage system (Figures 5 and 6) was used in the postoperative period. In our experience, air leaks resulting from decortication usually resolved by the 2nd postoperative day in many of these patients, as we have already published [10]. A formal system for postoperative pleural drainage is not readily available in Cameroon and our hospital was not equipped with a wall suction device. Figures 7 and 8 show some intraoperative specimens.

All the patients were transfused with 2 to 4 units of blood during surgery because of hemoptysis complicated by anemia and decortication which can lead to significant blood loss. None of our patients were placed on the respirator during the postoperative period.

### 2.4. Postoperative Management

Patients were transferred to the surgical intensive care unit overnight where they received standard postoperative care including intravenous fluids, analgesics, deep vein thrombosis prophylaxis, and postoperative antibiotics (ceftriaxone and metronidazole for 5 days). Chest physiotherapy was initiated as soon as possible. We aggressively managed any postoperative pain. The chest tube was removed after cessation of air leakage and drainage of less than 25 ml for 24 hours. A chest radiograph was performed before and after the removal of the thoracic drain.

Histopathological examination of the surgical specimen was performed. Anti-TB medication was administered for 6 months as recommended by the World Health Organization if active TB was found in the specimen. No antifungal therapy was initiated. After discharge, patients were seen at 1 week, 2 weeks, 1 month, 3 months, 6 months, and yearly thereafter.

The study was approved by the ethics committee of the Faculty of Medicine and Biomedical Science and the National Ethics Committee of Cameroon and conformed to the tenets of the Declaration of Helsinki. Written informed consent was obtained from the patients whose images are enclosed.

Data analysis was performed using Epi info 7 (Center for Disease Control and Prevention, Atlanta, GA).

## 3. Results

We analyzed the records of 20 patients (17 males and 3 females (sex ratio, 5.66); mean age, 30 years; age range, 23–65 years). TB was the only pulmonary preexisting disease in all cases. All the patients had undergone at least 6 months of treatment for TB (WHO regimen with rifampicin, ethambutol, isoniazid, and pyrazinamide) within the past 3 years. The HIV serology was negative in all patients.

Massive hemoptysis was present in 3 patients and moderate hemoptysis in 17. All presenting symptoms are shown in Table 1. Three patients with massive hemoptysis and one patient with right tension pneumothorax due to rupture of the aspergilloma were admitted and operated upon emergently. Complex aspergilloma was present in all 20 patients. Concerning the localization of the aspergilloma, the upper lobe was involved in all the cases with 14 (70%) present on the right. In a few cases, the aspergilloma involved two lobes (Table 2). The average preoperative hemoglobin was 8.2 g/dl (range: 6 g/L–11 g/L). All patients received blood transfusion (2 to 6 units) at the hospitals they were at before being referred to us.

All patients were managed surgically by means of a thoracotomy. Based on the localization, the thoracotomy was right posterior in 14 patients (70%) and left posterior in 6 patients (30%) (Table 3).

The surgical procedures are summarized in Table 3. In 3 patients, a right upper lobectomy was associated with the resection of the apical segment of the right lower lobe. Right pneumonectomy was performed in 2 patients and left pneumonectomy in 1 patient. The surgical findings were pachypleuritis, lung fibrosis and retraction, and mycetoma truffle in all cases. The aspergilloma was attached directly to the main bronchus in 3 patients.

Intraoperative complications were encountered in 9 patients, including bleeding that was readily replaced in 5 cases, massive air leak (2 cases) resulting from direct connection to the main lobar bronchus, and 2 cases of cardiac arrest due to arrhythmia while trying to dissect dense adhesions to the left side of the heart. All complications were successfully managed and no intraoperative deaths occurred.

The duration of surgery was between 120 and 220 minutes. The mean duration of chest tube drainage was 7 days (range: 4 to 12 days). The mean hospital stay was 7 days (range: 6 to 14 days). Postoperative complications were recorded in 4 patients (Table 4). We recorded one death: this was the patient operated for massive acute hemorrhage with severe chronic right heart failure who died one week after surgery following a successful left upper lobectomy.

The presence of the *Aspergillus* was confirmed in all specimens by pathological examination. Active TB was found at surgery and at pathology examination in 2 patients who were placed on anti-TB drugs as per WHO recommendations.

The mean follow-up period was 21.5 months (range: 12 to 40 months). Within this period, 3 patients were lost to follow-up after 1 year. The other 16 cases are still alive, yielding a survival rate of 80%. It is difficult to determine the incidence of restrictive lung disease. Survivors have resumed their usual activities. Many of them (60%) have resumed sport activities that they were not able to perform before surgery. There was no residual cough, fever, hemoptysis, dyspnea, or chest pain. The only pain resulted from chest wall scarring after thoracotomy since many of them developed hypertrophic scars (75%). None of the patients presented signs of recurrent disease.

## 4. Discussion

Although TB is a common respiratory infection in Cameroon, the prevalence of aspergilloma is unknown and no studies have been published on the treatment of aspergillomas colonizing former TB cavities. This is the first study which highlights the challenges of the surgical care of these patients in a setting where there is a lack of appropriate infrastructure and materials for such complex surgery. Complex aspergilloma was the only form encountered. We believe that the younger age of most of our patients and the lack of immunosuppression have contributed to minimize the number of complicated cases. Hemoptysis was universal in all our cases, and other authors have reported it as the most common symptom at presentation [3, 7, 8, 11]. Reported causes of hemoptysis are erosion of adjacent vessels, mechanical irritation of exposed vasculature in the cavity, release of endotoxin and trypsin-like proteolytic enzymes from the fungus, and the presumed superimposed bacterial infection [7, 12]. The prevalence of asymptomatic forms of PA has been reported to vary from 18 to 22% in previous studies [13]. The fact that all our patients were symptomatic can be explained by the poor access to health care and limited financial resources, leading to delayed diagnosis and treatment. This may also be associated with the high prevalence of complex forms; the patients may have sought medical attention only when they felt their lives were threatened. In fact, asymptomatic patients are rare in hospitals in Cameroon.

The treatment of pulmonary aspergilloma is still a subject of debate. Some surgeons believe that paucisymptomatic or asymptomatic patients can be managed conservatively with antifungal therapy and possibly arterial embolization [3, 8, 14]. However, interventional radiology has not yet been introduced to our country, and systemic antifungal therapy is usually ineffective and patients are unable to tolerate full therapeutic doses owing to toxicity [8]. The use of antifungal therapy requires a long period of follow-up with multiple plasma concentration tests to adjust doses [14]. This is difficult to achieve in a resource-limited setting such as our own. Furthermore, the new antifungal drugs (such as itraconazole) are not available in our country. We believe that, considering our patient and our environment, surgery is the most suitable and the only treatment modality. Thoracotomy and lung resection are effective, and nonoperative management should be considered only if the patient is at a high surgical risk or has severe pulmonary hypertension. This approach has been advocated by several authors [7, 8, 15–18].

The ideal surgical treatment consists of an anatomical resection involving the mycetoma and underlying cavity (a persistent cavity may result in recurrence). Therefore, we were inclined to choose either complete lobectomy or pneumonectomy if the remaining lobe were severely fibrotic and diseased. Cavernostomy may be useful in high-risk patients but was not performed in this series. Surgery was performed through a small skin incision (fully posterior, sparing the serratus anterior muscle) with no rib resection to minimize postoperative pain, stimulate a quick recovery, and achieve good cosmesis (as hypertrophic scars are very common in Cameroonians).

This procedure ranks amongst the most complex and difficult procedures in thoracic surgery owing to the presence of dense adhesions and pachypleuritis that may be very thick, ultimately necessitating decortication of the diseased lung. The surgery was a great challenge for us because of the type of PA we encountered (complex), the unavailability of double lumen endotracheal tubes, and the absence of a wall vacuum suction device to be used for drainage in the postoperative period.

Many authors have not found any benefit for additional antifungal treatment after surgical resection in immunocompetent patients [19, 20]. We did not perform intraoperative washout of the pleural cavity with amphotericin B or taurolidine as described by Farid et al. [15], nor postoperative irrigation with amphotericin B and neomycin similar to Chen et al. [8] to avoid postoperative empyema. After surgical resection, we performed a thorough pleural washout with saline and encountered only one case of postoperative empyema. Systematic culture of the specimen was not undertaken in our patients.

Postoperative complications occurred in four patients, two of which were taken back to the operating room owing to persistent bleeding and air leakage. Only one of our patients died (5%). This mortality rate is comparable to those previously reported (4 to 22%) [1, 8]. The acceptable morbidity and mortality rate in our study demonstrate than even in a resource-limited setting with financial and technical constraints, pulmonary resection for aspergilloma can be conducted with favorable results.

Although our sample size seems small, symptomatic aspergilloma is a rare disease in Cameroon and we see less than 10 cases every year. With an average follow-up of 21.5 months, at least 80% of the patients were alive. We use the term “at least” because we have considered patients lost to follow-up as dead. These patients may still be alive and in that case the survival rate would be higher. Farid et al. [15] found an actual survival of 60% at 5 years in a group of 18 patients operated on for complex aspergilloma. The survival at 1 year was 95% and all the patients were improved. Patients seen after 3 years were similarly doing fine and improved, including the 2 placed on anti-TB medication.

## 5. Conclusions

Although our series was small, this study represents the first published work on PA in our country and sub-Saharan Africa with long-term follow-up of surgical outcomes after a difficult procedure under difficult working conditions. Our results were favorable and comparable to those from previous reports, irrespective of our limited technical resources. We believe that aggressive surgical treatment must be considered the best option for symptomatic PA, especially given the unavailability of newly developed antifungal drugs in Cameroon.

## Figures and Tables

**Table 1:** Presenting symptoms of the patients.

Symptoms	Number (%)
Hemoptysis	20 (100%)
Cough	17 (85%)
Thoracic pain	15 (75%)
Dyspnea	12 (60%)
Chronic cough and sputum	6 (30%)
Weight loss	6 (30%)
Complex aspergilloma	20 (100%)

**Table 2:** Localization of the aspergilloma on the CT scan of the chest.

Localization	Number (%)
Right lung	14 (70%)
Left lung	6 (30%)
Upper lobe	20 (100%)
Upper lobe and middle lobe on the right	2 (10%)
Upper lobe and lower lobe on the left	2 (10%)
Total lung right	1 (5%)
Total lung left	1 (5%)

**Table 3:** Type of lung resection received as treatment.

Procedure	Number (%)
Unilateral lobectomy	12 (60%)
Double lobectomy	2 (10%)
Pneumonectomy	3 (15%)
Lobectomy and segmentectomy	3 (15%)

**Table 4:** Postoperative complications.

Type of complications	Number of patients	Treatment	Prognosis
Anemia	1	Transfusion of 3 units of blood	Good
Immediate postop bleeding	1	Taking back to the operating room the same day	Good
Bronchopleural fistula	1	Taking back to the operating room at day 4	Good
Postop empyema	1	Drainage	Good

## Abbreviations

HIV: Human immunodeficiency virus
PA: Pulmonary aspergilloma
TB: Tuberculosis
UHC: University Hospital Center.

## Data Access

Data and material used to write this paper are available on request.
